# Do CAD/CAM dentures really release less monomer than conventional dentures?

**DOI:** 10.1007/s00784-016-1961-6

**Published:** 2016-10-05

**Authors:** Patricia-Anca Steinmassl, Verena Wiedemair, Christian Huck, Florian Klaunzer, Otto Steinmassl, Ingrid Grunert, Herbert Dumfahrt

**Affiliations:** 1 University Hospital for Dental Prosthetics and Restorative Dentistry, Medical University of Innsbruck, MZA, Anichstr. 35, A-6020 Innsbruck, Austria; 2 Institute of Analytical Chemistry and Radiochemistry, University of Innsbruck, CCB, Innrain 80, A-6020 Innsbruck, Austria; 3 University Hospital for Cranio-Maxillofacial and Oral Surgery, Medical University of Innsbruck, MZA, Anichstr. 35, A-6020 Innsbruck, Austria

**Keywords:** Complete dentures, CAD/CAM dentistry, PMMA, Monomer release

## Abstract

**Objectives:**

Computer-aided design (CAD)/computer-aided manufacturing (CAM) dentures are assumed to have more favourable material properties than conventionally fabricated dentures, among them a lower methacrylate monomer release. The aim of this study was to test this hypothesis.

**Materials and methods:**

CAD/CAM dentures were generated from ten different master casts by using four different CAD/CAM systems. Conventional, heat-polymerised dentures served as control group. Denture weight and volume were measured; the density was calculated, and the denture surface area was assessed digitally. The monomer release after 7 days of water storage was measured by high-performance liquid chromatography.

**Results:**

Whole You Nexteeth and Wieland Digital Dentures had significantly lower mean volume and weight than conventional dentures. Baltic Denture System and Whole You Nexteeth had a significantly increased density. Baltic Denture System had a significantly smaller surface area. None of the CAD/CAM dentures released significantly less monomer than the control group.

**Conclusions:**

All tested dentures released very low amounts of methacrylate monomer, but not significantly less than conventional dentures. A statistically significant difference might nevertheless exist in comparison to other, less recommendable denture base materials, such as the frequently used autopolymerising resins.

**Clinical relevance:**

CAD/CAM denture fabrication has numerous advantages. It enables the fabrication of dentures with lower resin volume and lower denture weight. Both could increase the patient comfort. Dentures with higher density might exhibit more favourable mechanical properties. The hypothesis that CAD/CAM dentures release less monomer than conventional dentures could, however, not be verified.

## Introduction

Removable dentures are composite individual medical devices consisting of the denture base, denture teeth and, in some cases, additional components such as bonding agents or surface sealants. Until now, each denture was fabricated manually by a dental technician, resulting in an often very variable quality of prosthesis. The introduction of computer-aided design (CAD)/computer-aided manufacturing (CAM) manufacturing into removable denture prosthodontics now automatises many of the denture fabrication steps and is believed to produce a more constant denture quality.

The CAD/CAM denture fabrication workflow begins with the digitisation of the anatomical information. Then, the denture base and, in most cases, also the occlusion are designed virtually by using computer software (CAD). Following the digital design process, the denture base is milled fully automatised from prefabricated resin blocks (CAM).

In most CAD/CAM systems, the milling process produces denture bases with customised sockets for the insertion of the denture teeth according to the digitally designed occlusion. The denture teeth are then manually fixed into the sockets by using methacrylate-based bonding agents (Table 1). Baltic Denture System, however, follows a different approach: Instead of bonding the teeth to the denture base after milling, resin pucks already containing the teeth, which have been inserted to the material during the puck polymerisation, are being used.

**Table 1:** Overview over production specifics of different CAD/CAM denture systems

	Baltic Denture System	Vita VIONIC	Wieland Digital Dentures	Whole You Nexteeth
Milling localisation	Dental lab (milling centre planned)	Dental lab (milling centre planned)	Dental lab, milling centre	Milling centre
Digitalisation	Impression scan	Master cast scan or impression scan	Impression scan	Impression scan
Resin base	PMMA	PMMA	High impact PMMA	PMMA
Polymerisation record	“Tempering” (confidential)	Pressure > 200 kN, heat	Confidential	No information
Milling technique	Five-axe milling machine (two or three axes possible)	Variable	Five-axe milling machine (dry)	No information
Milling blank	PMMA puck with polymerisation-incorporated teeth	PMMA puck without teeth	PMMA puck without teeth	PMMA puck without teeth
Minimum base thickness	2 mm	2 mm	1 mm	1 mm
Mucosal surface finishing	Milling	Milling or manual finishing	Milling	Manual finishing and dip-coating
Oral surface finishing	Manual finishing	Manual finishing	Manual finishing	Manual finishing and dip-coating
Fixation of denture teeth to denture base	Polymerisation-related incorporation	Methacrylate-based bonding	Methacrylate-based bonding	Methacrylate-based bonding

While the mucosal denture surface is finished by milling in most systems, the oral denture surface mostly requires manual finishing and polishing. Whole You Nexteeth dentures are finished manually and then fully dip-coated by the manufacturer (Table 1).

One great advantage of the new fabrication protocols is the reduced number of patient visits for complete denture fabrication [1–3]. First evidences indicate that digitally fabricated dentures have a more favourable clinical outcome compared to conventionally fabricated dentures [3]. Besides the procedural advantages, digitally fabricated dentures are also hypothesised to have enhanced material-specific properties [1–5], because the denture base is milled from poly(methyl methacrylate) (PMMA) pucks that have been polymerised under high temperature and pressure [4]. The high pressure promotes the formation of longer polymer chains [6] and therefore leads to a higher degree of monomer conversion with lower values of residual monomer.

The presence of unreacted methacrylate monomer in denture base resins is undesired because it impedes the resin’s mechanical properties [7] and also compromises the product’s biocompatibility. Unfortunately, a certain amount of residual monomer is inevitable due to the monomer-polymer equilibrium necessary for free radical polymerisation of denture base resins [8], and the desired zero residual monomer cannot be achieved [8].

Besides influencing the resin’s mechanical properties, methacrylate monomer also leaches into the surrounding tissues and saliva [9, 10]. The released monomer is suspected of being responsible for allergic or chemical-irritative (cytotoxic) reactions to denture base materials [11, 12]. The resulting symptoms reportedly range from burning oral sensations [13], stomatitis [14] to edema and even ulceration of the oral mucosa [15].

In order to increase the biocompatibility [16], a maximal reduction of the residual monomer in denture base resins is desired. Since the amount of released monomer is proportional to the concentration of residual monomer within the resin [8, 17], different approaches for increasing the degree of conversion and thereby reducing the residual monomer content of denture base resins have been followed. Although different curing methods have been shown to lead to similar degrees of conversion in heat-polymerised resins [16], the application of higher pressure [6] or extended processing time [8] for polymerisation seem to be effective in enhancing the degree of monomer conversion. Another effective approach to reducing the residual monomer content is water bath post-polymerisation treatment [18].

As Table 1 illustrates, the PMMA pucks used for milling the denture bases of CAD/CAM-fabricated dentures are polymerised under high temperature and pressure, resulting in a supposedly highly condensed resin [4, 5]. Although the hypothesis that CAD/CAM-fabricated dentures should therefore release less monomer [4, 5] is conclusive, scientific evidences regarding monomer release from CAD/CAM-fabricated prostheses are missing so far.

### Aim

It has been the aim of this study to assess if CAD/CAM dentures do indeed release less methacrylate monomer than conventionally fabricated, long-time water bath heat-polymerised dentures. A secondary aim was to compare the denture weight, volume, density and surface area between CAD/CAM and conventionally fabricated dentures. In addition, the interrelations between denture volume, weight, density, surface area and the monomer release should be assessed in order to detect potential sources of monomer release.

## Materials and methods

### Specimens

Ten different consecutively numbered patient-generated master casts of edentulous upper jaws provided the basis for the specimen fabrication (Table 3). From each of the ten master casts, dentures were generated by using three different CAD/CAM denture fabrication systems (in alphabetical order: Baltic Denture System, Merz Dental GmbH, Lütjenburg, Germany; Whole You Nexteeth, Whole You Inc., San Jose, USA; Wieland Digital Dentures, Wieland Dental + Technik GmbH & Co. KG, Pforzheim, Germany/Ivoclar Vivadent AG, Schaan, Liechtenstein). Vita Zahnfabrik (Bad Säckingen, Germany), a manufacturer of components for open CAD/CAM denture systems (Vita VIONIC), provided CAD/CAM-fabricated dentures based on the master casts 1, 2, 4 and 5.

The CAD/CAM-fabricated dentures were all milled from PMMA-based resin loads (Table 1) and produced in minimum material thickness, bearing 14 teeth. The denture teeth were fixed to the denture bases by the manufacturers, according to each system’s standard protocol (Table 1). The dentures were delivered without previous water storage, in order to avoid uncontrolled monomer dilution.

In order to represent the clinical situation realistically, the specimens were manually finished by the same trained dental technician to high lustre on the oral surface. Only the Whole You Nexteeth dentures were not processed after delivery, because they are being delivered fully finished with overall surface coating. The other dentures were finished on the oral surface, following a standardised finishing and polishing protocol: When necessary, excess resin was removed by using cross-cut hard metal burrs. Elaborate and non-standardisable anatomical features, such as juga alveolaria and rugae palatinae, were waived. The primary surface refinement was performed by wet grinding with water-resistant sand paper (600 grit). The high-lustre polishing was performed by using wetted pumice and finally polishing paste (Edelweiss, Dentaurum, Ispringen, Germany). The mucosal denture surface was left unfinished, as intended by the manufacturers, and the dentures were stored dark and dry until the beginning of the chemical analysis.

Ten conventionally fabricated dentures, manufactured from the same ten master casts, served as control group. The conventional dentures were made from Candulor Aesthetic Red heat-polymerising resin (Candulor AG, Glattpark, Germany), polymerised in a long polymerisation cycle (75 °C water bath for 8.5 h, subsequent cooling of the mould in the water bath for 6 h). The dentures were finished and polished following the same previously described protocol performed by the same dental technician and then stored under the equal dry and dark conditions until the beginning of the chemical analysis.

### Determination of denture weight, volume and density

Prior to the chemical analysis, all samples were put into a desiccator for 12 h, in order to equal out different concentrations of surface water, which may have occurred due to different transportation conditions. Following the desiccation, each specimen was weighed and immersed in 200 ml of deionised water. The volume of the samples was determined by assessing the water displacement, and the denture density was calculated from the measured parameters.

### Chemical analysis

Following the physical measurements, the water-immersed dentures were stored in a Thermoshaker (Gerhardt, Königswinter, Germany) at 37.0 °C and 65 rpm for 7 days. The beakers, in which the dentures were soaked, were sealed with parafilm, in order to prevent the vaporisation of water and other volatile compounds. The seal also protected the solution from contamination by foreign particles.

After 7 days, the samples were removed, and the immersion fluid was extracted with 10 ml hexane three times. The organic phase was analysed regarding its monomeric content by using a HP 1090 LC liquid chromatograph (Hewlett Packard, Palo Alto, USA). The non-polar ACE C18 column used for analysis had a length of 150 mm and an inner diameter of 4.6 mm (ACE, Aberdeen, Scotland). The particle size was 5 μm.

In order to determine the best column flow, the Van Deemter plot curve was recorded and a flow of 0.8 ml/min was determined as most suitable. A solvent agent comprising water and acetonitril in a ratio of 1:1 in isocratic elution mode was used. The individual analytes were detected by using a diode array detector at 210 nm. Eight external standards of different methyl methacrylate concentrations (0.5, 1.0, 2.5, 5.0, 7.5, 10.0, 15.0 and 20.0 ppm) were prepared for calibration. The concentrations of the standards were chosen to fit the concentration of methyl methacrylate in the samples. The peak in question showed at a retention time of around 4 min. The concentration of released methacrylate monomer was determined six times for each specimen.

For the quantitative interpretation of the high-performance liquid chromatography (HPLC) chromatograms, a calibration curve was recorded. The known concentrations were then plotted against the peak areas and fitted with a line, which was forced to cross zero. The linear equation obtained was *y* = 39.344*x*. In this equation, “*y*” represents the peak area (mAU min) and “*x*” corresponds to the concentration (ppm). The regression coefficient (*R* ^2^) was 0.9972.

The concentrations of released methacrylate monomer were calculated by using the aforementioned linear equation.

### Quality management of chemical analysis

Intra-day precision, inter-day precision and recovery, determining how much of an added concentration is retrieved, of the method were measured for validation. The measurements were also verified by gas chromatography (GC).

Each chromatogram was evaluated on its own, in order to ensure that the automatically created baseline fitted the peak. If this was not the case, the baseline was redrawn manually.

### Assessment of specimen surface area

Both the palatal and the oral surface of each finished denture were scanned by using a 7Series Dental Wings scanner (Dental Wings Inc., Montreal, QC, Canada), which provides a precision of 15 μm, according to the manufacturer. The resulting digital data (3D meshes) was processed in STL format. After standardised cropping of the meshes, the specimen surface areas were determined by using the reverse engineering software GOM Inspect (GOM, Braunschweig, Germany).

### Statistics

The data was processed by using SPSS Statistics 22 (IBM, Armonk, NY, USA). Dentures generated from the same master cast were compared with each other. Descriptive statistics was used for calculating mean values and standard deviations. Statistical differences between specimens generated from the same master cast were evaluated by using the paired samples *t* test. Correlations between two scale variables were analysed by using Pearson correlation. The significance level was set at *α* = 0.05.

## Results

A total of 44 specimens was analysed; the specimen volume, weight and density are illustrated in Table 2; the denture surface area is illustrated in Table 3. Vita VIONIC dentures had a statistically significantly higher mean denture weight (*p* = 0.04) than conventional dentures; Whole You (*p* = 0.03) and Wieland Digital Dentures (*p* = 0.04) had a statistically significant lower mean weight than conventional dentures. Regarding denture volume, both Whole You and Wieland Digital Dentures had a statistically highly significantly lower mean denture volume (*p* < 0.01). Vita VIONIC had a higher mean denture volume, but the association fell short from being statistically significant (*p* = 0.06). The denture density varied between 1.25 g/ml (conventional denture) and 1.92 (Wieland Digital Denture) and was statistically significantly increased in Baltic Denture System dentures (*p* = 0.045) and statistically highly significantly increased in Whole You dentures (*p* < 0.01).

**Table 2:** Denture volume, weight and density

Denture origin	Conventional dentures			Baltic Denture System			Vita VIONIC			Whole You Nexteeth			Wieland Digital Dentures		
	vol (ml)	wt (g)	*ρ* (g/ml)	vol (ml)	wt (g)	*ρ* (g/ml)	vol (ml)	wt (g)	*ρ* (g/ml)	vol (ml)	wt (g)	*ρ* (g/ml)	vol (ml)	wt (g)	*ρ* (g/ml)
Cast 1	18.0	25.2	1.40	13.0	19.4	1.49	20.3	27.9	1.37	13.1	18.0	1.37	13.2	18.0	1.37
Cast 2	13.4	18.4	1.38	15.8	23.4	1.49	17.9	24.3	1.36	14.2	19.0	1.34	9.5	16.2	1.71
Cast 3	13.2	16.5	1.25	15.0	24.0	1.60				12.9	17.3	1.34	9.8	18.8	1.92
Cast 4	12.3	17.8	1.45	14.9	19.6	1.31	18.9	28.2	1.49	10.0	14.8	1.48	13.8	18.8	1.37
Cast 5	11.1	14.9	1.34	11.0	15.5	1.40	12.3	19.1	1.56	11.3	16.8	1.49	9.9	14.8	1.50
Cast 6	15.9	20.6	1.30	14.3	19.2	1.35				11.8	17.6	1.50	14.7	20.8	1.42
Cast 7	15.6	20.1	1.29	12.9	18.3	1.42				13.1	19.2	1.47	10.1	14.6	1.44
Cast 8	18.4	23.3	1.27	20.3	27.5	1.35				12.9	19.3	1.49	14.6	18.5	1.27
Cast 9	20.5	25.9	1.26	21.3	28.2	1.32				11.9	17.6	1.48	12.7	16.7	1.31
Cast 10	16.8	23.0	1.37	14.3	20.8	1.45				12.3	18.4	1.50	14.8	19.8	1.33
Mean (SD)	15.5 (3.0)	20.6 (3.7)	1.33 (0.07)	15.3 (3.2)	21.6 (4.1)	1.42 (0.09)	^a^	^a^	^a^	12.4 (1.2)	17.8 (1.3)	1.45 (0.07)	12.3 (2.2)	17.7 (2.1)	1.46 (0.20)

*vol* denture volume, *wt* denture weight, *ρ* denture density

^a^Mean values and standard deviations omitted because of smaller sample size

**Table 3:** Master cast dimension, monomer release and denture surface area

Denture origin	Tray size (intertuberal distance)	Conventional dentures		Baltic Denture System		Vita VIONIC		Whole You Nexteeth		Wieland Digital Dentures	
		MMA (ppm)	Surf (cm^2^)	MMA (ppm)	Surf (cm^2^)	MMA (ppm)	Surf (cm^2^)	MMA (ppm)	Surf (cm^2^)	MMA (ppm)	Surf (cm^2^)
Cast 1	L (45 mm)	<0.2	112.6	1.6	95.8	8.2	120.7	4.3	102.9	7.9	103.7
Cast 2	L (47 mm)	2.4	116.8	0.6	119.4	1.1	123.7	5.4	134.9	<0.2	120.5
Cast 3	L (44 mm)	1.4	103.0	0.6	103.7			10.0	113.7	3.7	100.3
Cast 4	M (42 mm)	1.3	83.9	0.3	79.0	12.6	86.3	6.6	90.5	4.1	85.0
Cast 5	M (42 mm)	<0.2	83.0	0.3	77.0	5.2	87.3	10.9	97.1	4.9	84.3
Cast 6	L (47 mm)	1.9	99.8	0.4	88.0			2.2	103.0	1.1	99.8
Cast 7	L (46 mm)	1.4	106.8	0.4	96.2			4.1	118.2	0.8	104.8
Cast 8	XL (50 mm)	<0.2	109.5	0.7	112.6			5.0	124.9	7.2	108.5
Cast 9	XL (54 mm)	5.6	134.5	0.4	129.2			7.3	145.7	10.0	131.4
Cast 10	XL (53 mm)	0.6	109.8	0.9	98.4			4.4	117.5		107.9
Mean (SD)		1.5 (1.6)	106.0 (15.1)	0.6 (0.4)	99.9 (16.8)	^a^	^a^	6.0 (2.7)	114.8 (17.2)	^a^	104.6 (14.3)

*MMA* monomer concentration, *Surf* denture surface area

^a^Mean values and standard deviations omitted because of smaller sample size

The dimension of the denture surface area varied between 77.0 and 145.7 cm^2^. Baltic Denture System prostheses had a statistically significantly smaller surface area than conventional dentures (*p* = 0.02); Vita VIONIC and Whole You Nexteeth dentures had a statistically significantly increased mean denture surface area compared to conventional dentures (*p* = 0.02 for Vita VIONIC, *p* < 0.01 for Whole You Nexteeth, Table 3). The difference in mean denture surface area between Wieland Digital Dentures and conventional dentures was not statistically significant.

The monomer measurement protocol showed a HPLC recovery rate of 94.7 %. The accordance between HPLC and GC was 99.4 %. The Wieland Digital Dentures sample generated from master cast 10 was used as a reference for another analysis. Therefore, the amount of methacrylate released from this specimen has not been measured.

All dentures released only very little monomer during the 7 days of water storage. Three of the conventionally fabricated dentures and one Wieland Digital Dentures prosthesis had a very low monomer release below the basal white noise of the HPLC. Table 3 presents the methacrylate monomer released by each specimen.

**Fig. 1:**
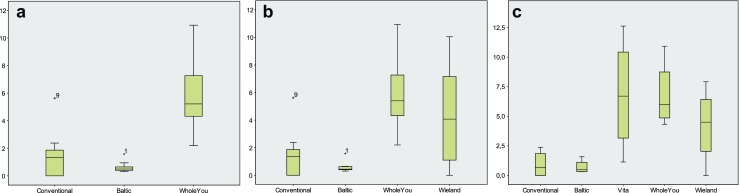
**a**–**c** Monomer release (ppm) of dentures fabricated from the master casts 1 through 10 (**a**), 1 through 9 (**b**) and 1, 2, 4 and 5 (**c**)

The full number of ten samples was analysed from Baltic Denture System, Whole You Nexteeth and the conventional dentures (Fig. 1a). Baltic Denture System dentures released the least monomer (mean = 0.6 ppm, SD *= 0.4*), but the difference was not statistically significant. The conventional dentures had a higher monomer release (mean = 1.5 ppm, SD = 1.6). The monomer release from Whole You Nexteeth dentures was statistically highly significantly increased compared to conventional dentures (*p* < 0.01), with a mean of 6.0 ppm, SD = 2.7.

Also, among the nine specimens based on the master casts 1 through 9 (Fig. 1b), which were provided from all companies except for Vita, the monomer release was smallest among Baltic Denture System dentures (mean = 0.6 ppm, SD = 0.4) and among conventionally fabricated dentures (mean = 1.6 ppm, SD = 1.7). Whole You Nexteeth dentures released a mean of 6.2 ppm monomer (SD = 2.8), and Wieland Digital Dentures released a mean of 4.4 ppm (SD = 3.4).

Among the specimens generated only from master casts 1, 2, 4 and 5 (Fig. 1c), the conventional dentures released a mean of 1.0 ppm monomer (SD = 1.1), Baltic Denture System dentures released a mean of 0.7 ppm (SD = 0.6), and Wieland Digital Dentures released a mean of 4.3 ppm (SD = 3.2). Vita VIONIC dentures released a mean of 6.8 ppm monomer (SD = 4.9), same as Whole You dentures (mean 6.8 ppm, SD = 2.9). The range of monomer release was smallest among Baltic Denture System dentures and among conventionally fabricated dentures (Fig. 1c).

There was no statistically significant correlation between monomer release and denture weight or volume. Subsequently, there was also no statistically significant correlation between the denture density and the methacrylate monomer release. A statistically significant correlation between monomer release and denture surface area could not be shown for the CAD/CAM-fabricated dentures, but the correlation between denture surface and methacrylate monomer release fell short from being statistically significant (*p* = 0.05).

## Discussion

### Experimental set-up

The present study is the first to evaluate the cumulative monomer release from whole complete dentures. While monomer measurements from standardised specimens [19, 20] is suitable for comparing different denture base resins or processing methods, the design of the present study enables the determination of the clinically more relevant monomer release from the customised medical device “complete denture”.

Heat-polymerised resin was used for the reference samples, because it has been shown that heat-polymerised resins have more favourable chemical properties than autopolymerised resins [21]. All dentures, except for the fully coated Whole You Nexteeth dentures, were finished manually on the oral surface by a dental technician. The finishing included high-lustre polishing and was performed following a standardised protocol, since the degree of surface refinement is another factor influencing the monomer release [22]. Nevertheless, manual processing, even when performed by the same and experienced technician, can never provide full standardisation, as for instance, the contact pressure during polishing or the moisture penetration of the pumice can realistically never be fully controlled. This may result in minimal variations of the surface conditions. The dark storage protected the residual of the denture resin from light-induced degradation.

### Physical denture properties

The observation of the great variability in denture weight and volume seems surprising at first. As Table 3 shows, the master casts purposely represented maxillary jaws of very different dimensions. The differing minimum material thickness given by the manufacturers provides one explanation why the denture weight and volume differ between dentures of different brands fabricated from the same master cast. To be satisfactory, this explanation would require analogous distributions of the denture volume and weight between the different manufacturer groups. This was not the case. Perhaps the determination of the functional denture border might be responsible for the persisting differences.

Although the denture surfaces seem to correspond better with the master cast size, the variance in denture surface is still considerable. Through the high resolution (15 μm) of the denture surface scanner, the surface finishing has found some consideration. The denture base thickness, on the other hand, contributes only comparatively little to the denture surface.

The most probable explanation for the great variability is the different vestibular and posterior extension of the denture bases. The differences could even be detected visually. The reason for the varying border extension of the specimens is that the digital denture base design is not performed fully automatised, but the border points have to be located and defined manually.

Own clinical observations from patient treatment with CAD/CAM dentures support this hypothesis: Even dentures fabricated by the same manufacturer from the same impression can differ in fit and retention in the patient. These findings indicate that although the CAD/CAM fabrication process provides a higher degree of standardisation of the medical device complete denture, there is still a certain extent of variability. Absolute standardisation will not be possible as long as human input is involved in the manufacturing process.

Nevertheless, a thinner minimum material thickness can be advantageous, particularly in the palatal plate area, where a thinner denture may increase the patient comfort. Although further investigations will be necessary for evaluating if the industrially processed, thinner CAD/CAM dentures exhibit equal physical properties to conventional dentures, the significantly increased material density found in Baltic Denture System dentures and WholeYou Nexteeth dentures might suggest a higher degree of condensation, lower resin porosity and therefore increased fracture toughness.

### Chemical analysis

The suitability of HPLC analyses for determining denture base residual monomer has previously been demonstrated [23], and the validity of the results has been ensured by dual measurement of monomer release (HPLC and GC). The duration of water bath immersion of 7 days was chosen in analogy to similar studies [19]. Hexane was chosen as extracting agent, because it shows good phase separation with water, because it was compatible with the planned subsequent analytical methods and because it was also determined as suitable in similar studies [19, 24].

### Monomer release

Overall, the monomer release from all specimens was much lower than reported for standard specimens in similar studies [19, 24]. The cut-off value for biocompatibility of denture base resins seems to be 1–3 % residual monomer [25], and the ISO 1567 standard permits a maximal residual monomer content of 2.2 % wt (equalling 22,000 ppm) [26]. However, it must be stated that the experimental setting of the ISO standard is completely different (specimen design and surface, solvent agent type, volume and conditions). A direct comparison of the results is therefore impossible. Nevertheless, the tested denture base resins are commercially available and therefore should be CE-certified. To reach certification, the companies are required to prove the product’s compliance with the ISO standard. It may therefore be deducted that the evaluated dentures are biocompatible.

The very small range of monomer release among Baltic Denture System dentures is an interesting finding indicating a high degree of cross-linkage. The manufacturer claims to use a special procedure referred to as “tempering” in the production of the PMMA loads. The details of the tempering process, however, are not revealed. Possibly, this process serves to deplete the residual monomer effectively and to a high degree. Although the mean value of monomer release was higher in Whole You Nexteeth dentures in the present study, it has to be stated that (1) the effective amount of released monomer was still very low and (2) the dentures provided for the present study were purposely not immersed in water, as it is the usual custom during denture delivery of Whole You Nexteeth dentures. The role of the Whole You Nexteeth’s dip-coat in methacrylate monomer release remains unclear, since other studies have shown that surface-coated heat-polymerised denture resins release less monomer than non-coated specimens [22]. Since the chemical composition of the dip-coating is not revealed by Whole You, Inc., speculations on the reason of the observed difference are futile. The manufacturer, however, claims that the dip-coat is not PMMA-based.

It may seem surprising that there was no statistically significant association between the denture volume or denture weight and the cumulative monomer release. Furthermore, the dentures with the lowest cumulative monomer release (Baltic Denture System) were among the dentures with the highest weight and volume, and the dentures with the lowest mean weight and volume (Wieland Digital Dentures, Whole You Nexteeth) released more residual monomer. Two possible explanations come to mind: On one hand, it is possible that even industrial polymerisation protocols cannot achieve a constant polymerisation degree. Recent studies have found that only the superficial layer of the denture base resin deteriorates during usage [9]. Besides, monomer also diffuses into the surrounding mould during the polymerisation process [8]. It is therefore very likely that a lot of residual monomer was lost to the water-immersed mould during the polymerisation process of the conventional dentures which served as control group. The CAD/CAM denture bases, on the other hand, are milled from industrially polymerised, disc-shaped PMMA loads. The loads are required to have dimensions that exceed the exterior borders of denture bases. While the industrial production process may enable a constant product quality, the high material thickness of the PMMA load might also hinder the diffusion of methacrylate monomer from the centre of the load, which often ends up being the surface of the milled denture. Consecutively, it could be hypothesised that monomer does perhaps not effuse from the inner resin core of dentures with greater thickness. Another conclusive explanation might be that the bonding agents used for fixing the teeth to the milled sockets are a relevant source of methacrylate monomer release. All CAD/CAM denture systems except for Baltic Denture System use methacrylate-based bonding agents, and the distribution of the bonding agent within the milled denture base socket may not always be homogenous.

**Fig. 2:**
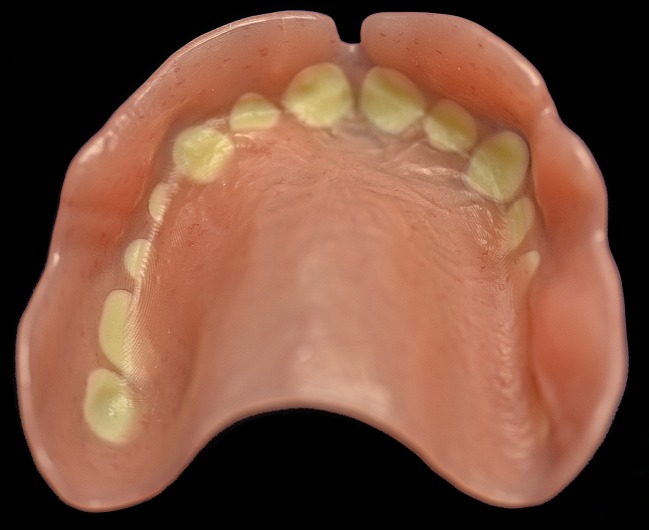
Communication of bonding gap with mucosal denture surface

This might be of clinical relevance: If a defined amount of monomer is released from the whole denture base, the local surface concentration of monomer is much lower than if the bonding agent within the (very localised) adhesive gap is the main monomer source. The bonding gap can directly touch the alveolar mucosa if patients have only little available vertical space (Fig. 2). If the measured monomer release originates mainly from these very small areas, the local surface monomer concentration on the mucosal surface of the bonding gaps would be expected to be rather high. This could bear a considerable risk of local mucosal irritations, focused on the mucosal contact areas.

Another aspect supporting the hypothesis that the bonding agent contributes substantially to the monomer release from CAD/CAM dentures is that the expected statistical correlation between denture surface and monomer release was strongest in the conventional dentures, in which the denture teeth are polymerised into the base. In the Baltic Denture System dentures, which follow the same tooth fixation protocol, a possible statistical association between denture surface and monomer release might be obscured by the aforementioned tempering of the dentures.

While fully standardised experimental set-ups, as used for instance in the ISO standard, are necessary to ensure comparability of different resin types, complete dentures are individual composite medical devices. For determining the clinically relevant monomer release from these medical devices, an experimental set-up considering all involved components is necessary. The present study suggests that although the automatised production may standardise the manufacturing process to a certain degree, CAD/CAM-fabricated dentures are still not standardised medical devices.

### Clinical implications

CAD/CAM denture fabrication has numerous advantages. It enables the fabrication of dentures with lower resin volume and lower denture weight. Both are desirable features as they can increase the patient comfort. Dentures with higher density might exhibit more favourable mechanical properties.

The present study shows that CAD/CAM dentures release very little monomer. However, CAD/CAM-fabricated dentures do not release statistically significantly less monomer than manually fabricated, water bath long-time heat-polymerised dentures. A statistically significant difference of cumulative monomer release might nevertheless exist in comparison to other, less recommendable denture base materials, such as the frequently used autopolymerising resins. The source of the observed monomer release needs to be explored in further studies.
